# Supplementary material for: Patterns of Intron Gain and Loss in Fungi
Source: PLoS Biol. 2004 Nov 30;2(12):e422. doi: 10.1371/journal.pbio.0020422 (PMC532390; doi:10.1371/journal.pbio.0020422)
Supplement: Table S1 — Also available at http://genes.mit.edu/NielsenEtAl/. (4.3 MB ZIP). [file pbio.0020422.st001.zip › NielsenEtAl/html/1292.html]

AN2927.1.NCU00978.1.MG10826.1.FG01137.1


```
 CLUSTAL W (1.82) Multiple Sequence Alignments - Introns Inserted


Sequence 1: NCU00978.1	918 aa
Sequence 2: FG01137.1	884 aa
Sequence 3: MG10826.1	905 aa
Sequence 4: AN2927.1	828 aa
Alignment Length: 993 aa
Number Identitical Residues: 246 aa
Alignment Score (without introns) 12495


MG10826.1 	-MSIVSPTPMGGS-QFTTLGRRTSTRQALRRPPSR---QQLSHRPTSDSNPIGSSRATDS
NCU00978.1	-MSSISPTPMGGGSHFTSMGRRTSARQALRRPPSR---PMLNR---SESNPQSQSQQYSN
FG01137.1 	-MATASPTPMGNG-----LVRRTSQRQALRRLPSR---PAANH---NESFSAPTNQPLPN
AN2927.1  	MASPNSLSTAHATALHQQQYRQMSRASSVSRSGSRRSSPEVHNSVALRSNAASISKQYSA
          	  :  * :.    :      *: *  .:: *  ** ::    .  :  * .   .:    

MG10826.1 	NLQGGAQKYVNDNDSSDEEIPVPMKLSALTKALLDDGGPS---------AAAANAAQPAA
NCU00978.1	-----------LNDSSDDEIPVPMKLSALTKALLNDGHASEASSFAQHGAPEDLQLSPPA
FG01137.1 	GYH--PKPQQFHDDSSEDEIPVPMKLSALTKALLNDGDP----------APAERPVSPP-
AN2927.1  	G------------DSSDDEVPEP-KFSASVKALLDEDDGN---------------ASPR-
          	.            ***::*:* * *:** .****::.  .                .*  

MG10826.1 	IPTRNMSYLDRGLEHKDSEREVS-TLRESRRNVRAGSAQLTSSPNR---------GVEPS
NCU00978.1	RPPSRVTRRSALLSSKSEERSEGVRAREARRSIRGGSLQPMDRPTS-PVQNDSSNNRDGS
FG01137.1 	----RTRRRTSALNASTSSTS------ETRRQRRSGSVQPHDTRSSRPTSPAQVQSREIS
AN2927.1  	-------LRTRGLN-------------EYRPNLRVGSAASSN------------------
          	            *.             * * . * **    .                  

MG10826.1 	SAPRKRVVRLSSTNVSVNTTPGGSSLQSKVRRSSTHKRAPSKDRTDERNKVSGPEPVVSQ
NCU00978.1	PVPRKRVVRLSNPVQGPVSFSG--NVRRLSSTTANQRRTRTESR--EREVEKEIPSFLQQ
FG01137.1 	PV-RKRVVRLSNTPQSLNQLKP--TKRRSTSTSRSTQRSRPQSRPDSRDMSSDERYDSKQ
AN2927.1  	-------ERRSRTTSPLDQSNG--SPAPRVVRIGSVLGSGSKLRREGSHLTSGENAEVEA
          	        * * .           .         .   : .: * .  .  .      . 

MG10826.1 	DS-TAAADSVAGINTPVSGARRVRIVSNSSATRGR----THGSTLRSSSSAGTSRLPSSA
NCU00978.1	QG-DAGSDSAGDAQTPVI--RTVRIAVGSGSSGSKGRISLGGGGGSSGSSFAGVSGKRGT
FG01137.1 	DSKDSKQDSQLDVNTPAQGGRVVRINAGSPSNRSR----LGSTGPSSGRSADRSFVDRST
AN2927.1  	EA----KHYSSDFITPGPRTRSVRISTSRTHTRSP-----TSASPSGRRSASRSPLHEEG
          	:.     .   .  **    * ***  .   . .       .    .  *          

MG10826.1 	AHSDHEQPEEPRTEHRTRTSLNN------SSGANTARKPDDAAPQSLMRMKRIGQLPGSF
NCU00978.1	GYSDHENPEDPATIGR-------------PQTVAP---------QSSMRIKRVAKLPGSF
FG01137.1 	-IEDNDHPEEPATVARNPPPFHHGSVSRYPSTTTRTRPEDNGNLQNSMRVRRVSKVAGSF
AN2927.1  	---SADRPEEEGKSRYDDYAPIIG-----TSSVLRSRNPEDIGIQSSLRVKRVGRLTGTF
          	   . :.**:  .      .   .     .. .  :   ..   *. :*::*:.::.*:*

MG10826.1 	LSGPARRGR-RRQSDEEENAPADEQYPDQYMHGLREHEPESIARHQPGSGAGSSFRLSSY
NCU00978.1	LSGPARRGR-RRQSDEDEAAHGENE-------GMG-MNQDNDGNYESHMQSHVEAAMGEQ
FG01137.1 	LSGPARRGR-RRQSEEEGEGHADGD-------ILSSHEADGQPAEDQHAASYYGDGIRDF
AN2927.1  	LNGPARRGVLRRQSEEDQSPSYLSDSKE----AEGGDNNDYNYKSARASSPKVSWADPEQ
          	*.******  ****:*:       :  .         : :          .       . 

MG10826.1 	RDQAAASGSPVSIKDPARAALRNKTSPGSSAKTDAETNKE--------------NVPPEE
NCU00978.1	HLS--FYGSNNNYRDYAASGSPVSGKAGAVRR--RESNFR------------TMERDNGE
FG01137.1 	NSGSPVSGQAAARAIHRRNASNIDLRLGAARQYSRETSPRESTPEVQVPEPEAKPEPEEE
AN2927.1  	PQKTTDYRRISSGDGPFSRSSSPKSYVSHSKSTPGSSSDAS-----------SKPSSSKE
          	   :.              .   .   .       .:.  .           :      *

MG10826.1 	AHEPAPVFQPVLPSALDQENAVSAAVARSKEVISMHEKLASRDPAPLPQVQAPVIPKQR-
NCU00978.1	LGYKIAEPHPDMPMNNDKENEAPPTFKRAK--LTPSAMLDHEIPKPVIPEPMALDIGRDI
FG01137.1 	IHYRLPAPRPELPSGRDQENDQP-SLKRNK--PSMDAILDKMPKRPMSADPAPVRPS---
AN2927.1  	PIFKVPPP-PTLPSTRDQENEPPPTFKRTK--PQGFDFLDKPEKLSVVYGDDKKDQELP-
          	     .   * :*   *:**  ..:. * *        *      .:             

MG10826.1 	-------APSPDRKVLSHMSRNTPLRPAPPPPPKMSVLDAVTSTAGAATTAQAIKKQRVL
NCU00978.1	GRDLKRLSISPDRKALAHKSQNTPHRPAPPPPPKMSVVETATAAAGASATAGAAKKRQVL
FG01137.1 	---------SPDRKPLASMARNTPLRHAPPPPPKMSVLEAATATAGAATTT-QAKQRRNI
AN2927.1  	-------AGTSPRKILSNRSNNTPHRPAPPPP-KMSVLETATATGGAATAS-QSRKKRSQ
          	       : :. ** *:  :.*** * ***** ****:::.*::.**::::   ::::  

MG10826.1 	LKVNGRAYNRLDCIGRGGSAKVYRVTAENGKMFALKRVSLENADTTAVKGFLGEIELLRR
NCU00978.1	LRVNGRTYTRIDCIGRGGSGKVYRVSAESGKMLALKRVSLEHADENTVKGFKGEIDLLKR
FG01137.1 	LRVNGVAYTRLDCLGRGGSGKVYRVAAENGKMFALKRVSLENADDSTIKGYLGEIDLLKK
AN2927.1  	VSINHKPFTRLDCIGRGGSSRVYRVMAENYKIFALKRVNLEDVDPTTLAGYKGEIDLLKK
          	: :*  .:.*:**:*****.:**** **. *::*****.**..* .:: *: ***:**::

MG10826.1 	LSGVDRVIQMYDSELNQEKKLLSV0LMEMGELDLGDLLKSR-HSQEDYKLDTVFVRFIWK
NCU00978.1	LNGVERVIQLIDHELNLEKQVLSV0LMEVGELDFNTLLKSRQSTAEGARLDPTFIRYYWK
FG01137.1 	LGEVERVIKLFDCEMNTEKQVLTL0LMEIGELDFNTFLRKR-YNPEAAKFDPVFVRFYWK
AN2927.1  	LENVDRVVRLFDWELNSDKRTLSV~LMEIGESDLEKVLTYK-LNAEDATFDINFTRFYWK
          	*  *:**::: * *:* :*: *:: ***:** *:  .*  :  . *   :*  * *: **

MG10826.1 	EMLECLQSVHACDIVHSDLKPANFVMVKGRLKLIDFGIAGAIQTDETVNVHRENQIGTPN
NCU00978.1	EMLECVQAVHARDVVHSDLKPANFVLVQGRLKLIDFGIANAIQTEMTVNVHRETQIGTPN
FG01137.1 	EMLECLQAVHQCDIVHSDLKPANFVLVKGQLKLIDFGIANAIQTDETVNVHRETQVGTPN
AN2927.1  	EMLECVQAVHEYNVVHSDLKPANFLLVQGRLKLIDFGIANAIQ-DNTVNVHREQQVGTPN
          	*****:*:**  ::**********::*:*:*********.*** : ******* *:****

MG10826.1 	YMSPESLMDSAHYSATAAN-------NGRPDRSLNRTRVMKLGKPSDVWSLGCILYQLVY
NCU00978.1	YMSPESLMDSNQYAFTSAQNGKFCIPPPLNHRQHGAPKLMKLGKPSDVWSLGCILYQMVY
FG01137.1 	YMSPESLMDSN---------------NPRGGRAPGRPKLMKLGKPSDIWSLGCILYQMVY
AN2927.1  	YMSPEALVDSN----------------ASLGLPASVGKMMKLGKPSDVWSLGCILYKMVY
          	*****:*:**                        .  ::********:********::**

MG10826.1 	GTPPFGHIPNQLARCQAIIAWDHKISFPEKGAGGVFVPPSLVRTMKRCLIRDQQYRPTCE
NCU00978.1	GLPPFGKIANQMSRCQAIINWSYQIDFPEATEDGSRVPPSVIRTMRRCLNREQRERPTCD
FG01137.1 	GLPPFGHIANQMSRCQAIINWDHEIEFPSRGMGGMSVPPSLIRTMRRCLHRDHHMRPNCE
AN2927.1  	GQPPFAKIAKYYERIMAIPNPKVKIDFPAFGVGGIQVPPGLIRTLKGCLQRDQTLRPTIS
          	* ***.:*.:   *  **   . :*.**    .*  ***.::**:: ** *::  **. .

MG10826.1 	ELLTETDPFLYPQEVD--------MDRVLPMTEELLARVIHSVVNRCRDRMPTDGELMTA
NCU00978.1	ELLADTDPFLYPQEFDPSVYAAAEHGRVLPVTEELLSRLIQSVVQRCKERMPTEAEVVNT
FG01137.1 	ELLHESDPFLYPAELT---------DKALPIDEELLGRIIQSVVTRCRERMPTEAEILSV
AN2927.1  	DLLGQRDPFLYPDAQL---------EGAVPITQDVLGRILVNVVNHCRVRGVPNDEELAA
          	:** : ******               .:*: :::*.*:: .** :*: *  .: * : .

MG10826.1 	WPNAYWASVKKSVGSKEARS--------------
NCU00978.1	WPQAYWATVRKAVMGAGNNAGGSGGGNGSGGHQR
FG01137.1 	WPQAYWSSVKKAMAGRM-----------------
AN2927.1  	WPAGFFAKIKAALEENP-----------------
          	** .:::.:: ::
```
